# Innovation in Hyperinsulinemia Diagnostics with ANN-L(*atin square*) Models

**DOI:** 10.3390/diagnostics13040798

**Published:** 2023-02-20

**Authors:** Nevena Rankovic, Dragica Rankovic, Igor Lukic

**Affiliations:** 1 Department of Cognitive Science and Artificial Intelligence, School of Humanities and Digital Sciences, Tilburg University, 5037 AB Tilburg, The Netherlands; 2 Department of Mathematics, Informatics and Statistics, Faculty of Applied Sciences, Union University “Nikola Tesla”, 18000 Nis, Serbia; 3 Department of Preventive Medicine, Faculty of Medical Sciences, University of Kragujevac, 34000 Kragujevac, Serbia

**Keywords:** hyperinsulinemia, ANN + orthogonal vector plans, ML algorithms

## Abstract

Hyperinsulinemia is a condition characterized by excessively high levels of insulin in the bloodstream. It can exist for many years without any symptomatology. The research presented in this paper was conducted from 2019 to 2022 in cooperation with a health center in Serbia as a large cross-sectional observational study of adolescents of both genders using datasets collected from the field. Previously used analytical approaches of integrated and relevant clinical, hematological, biochemical, and other variables could not identify potential risk factors for developing hyperinsulinemia. This paper aims to present several different models using machine learning (ML) algorithms such as naive Bayes, decision tree, and random forest and compare them with a new methodology constructed based on artificial neural networks using Taguchi’s orthogonal vector plans (ANN-L), a special extraction of Latin squares. Furthermore, the experimental part of this study showed that ANN-L models achieved an accuracy of 99.5% with less than seven iterations performed. Furthermore, the study provides valuable insights into the share of each risk factor contributing to the occurrence of hyperinsulinemia in adolescents, which is crucial for more precise and straightforward medical diagnoses. Preventing the risk of hyperinsulinemia in this age group is crucial for the well-being of the adolescents and society as a whole.

## 1. Introduction

The development and influence of risk factors in children and adolescents can have far-reaching consequences, potentially leading to the manifestation of various chronic non-communicable diseases in later life. In the initial stage of insulin resistance and hyperinsulinemia, there may be few obvious symptoms, and these symptoms may not be immediately apparent. These symptoms, such as fatigue, hunger, a decrease in concentration, and nervousness, can only become evident when glucose levels in the blood begin to increase. Over time, the progression of these conditions may result in an increase in body weight, hypertension, hyperlipidemia, the development of macrovascular diseases and neuropathies, and other serious chronic health issues [1,2,3]. Typical blood glucose values are from 3.8 to 5.5 mmol/L, and insulin from 2.6 to 24.9 μU/mL [4,5,6] In the case of insulin values at 0 min being greater than 15 μU/mL and insulin values after an oral glucose tolerance test (OGTT) being greater than 75 μU/mL at 120 min, the value of total insulin is more significant than 300 μU/mL, and hyperinsulinemia is diagnosed [7]. The growing prevalence of hyperinsulinemia in adolescents is a severe and global problem of modern times. Adolescence is a period of complete somatic, sexual, psychosocial, and emotional growth, which takes place from the end of childhood to adulthood [3,6]. During this period, changes in the field of insulin sensitivity can also be noted. Although the mentioned aspects are considered physiological, the influence of certain factors such as genetic predisposition, obesity, insufficient physical activity, environmental factors, inadequate nutrition, stress, and insulinemia values can be pathophysiological [8,9]. Insufficient verification of known factors despite possessing adequate knowledge thereof may give rise to potential risk factors associated with the pathophysiological condition under consideration. Particular importance should be given to the early identification of adolescents at risk of developing hyperinsulinemia [10]. An OGTT with insulinemia is a significant indicator of glucose metabolism disorders, that is, the ability to regulate blood sugar levels and insulinemia values of >15 μU/mL after and/or during the 120 min. Additionally, an OGTT value of >75 μU/mL is considered the threshold for diagnosing the presence of hyperinsulinemia [11].

In order to enhance efficiency, accuracy, and facilitate early prediction for swift and accurate medical diagnosis, it is imperative to seek support from artificial intelligence (AI) tools. The utilization of machine learning (ML) algorithms in this digital age is crucial for making data-driven predictions. By leveraging a diverse range of AI tools and ML algorithms, the risk factors causing hyperinsulinemia in adolescents can be rapidly and effectively detected. However, the traditional statistical approach to risk assessment is often laborious, as it not only requires a significant amount of time for the various necessary analyses, but also entails a lengthy process to analyze all the required parameters.

This study, therefore, attempts to contribute to the knowledge base by being the first that compares the most popular and commonly used ML algorithms with a proposed new methodology that will use ANN architectures constructed based on Taguchi’s orthogonal vector plans. Moreover, this paper has two main research goals. The first research goal of this paper is to examine the presented models using various machine learning algorithms and artificial neural network architectures based on different Taguchi’s orthogonal vector plans. Furthermore, we want to determine the most accurate approach to assess the risk of hyperinsulinemia in adolescents. The second research goal of this paper is to identify the most significant factors that contribute to the development of hyperinsulinemia in adolescents. This will involve analyzing the data to determine which variables have the greatest impact on the risk of hyperinsulinemia in this population.

The rest of the paper is organized as follows: Section 2 gives an overview of the current research for improving medical diagnostics and of different health-care predictions using statistical methodologies and ML algorithms. Section 3 describes the new model ANN-L for hyperinsulinemia diagnostics through the main steps of the robust design of the experiment, compared with most commonly used ML algorithms. Section 4 presents obtained results. Section 5 discusses the results. The concluding remarks are given in the Section 6.

## 2. Related Work

In this section, we want to discuss relevant, newly published studies that relate to our research. Moreover, we want to point out the ideas and goals of the authors, tackling the problem meant to be solved using medical data.

### 2.1. Naïve Bayes

The effectiveness of medical diagnosis heavily relies on the accuracy of data analysis and prediction. Previous research [12] has demonstrated that the naive Bayes algorithm is capable of delivering outstanding results, particularly when rule extraction is performed using the Pima diabetes dataset as input. The findings indicated that naive Bayes outperformed other machine learning (ML) algorithms in terms of accuracy. The results of the simulations in the study in [13] presented the effectiveness of the classification techniques in medical diagnostics such as naive Bayes and random forest. The authors in [14] proposed a strategy called feature correlated naive Bayes (FCNB) to detect positive cases at an early stage for COVID-19 treatment. Another study [15] stated that it is possible to predict intra uterine growth restriction during pregnancy with an accuracy of 84% using the naive Bayes classifier. In [16], based on the analysis of the 25 pieces of testing data from 105 pieces of training data, the researchers obtained a 96% accuracy of the naive Bayes classifier. 

### 2.2. Decision Tree

The authors in reference [17] presented a cutting-edge prediction model that leverages the synthetic minority oversampling technique, genetic algorithm, and decision tree (PMSGD) to classify diabetes mellitus in the Pima Indians Diabetes Database (PIDD) dataset. Another study [18], utilizing fuzzy logic and decision tree algorithms, achieved an accuracy rate of 88% in diagnosing heart disease. The research conducted in [19] presented two models, a Probabilistic neural network based on the dynamic decay adjustment and a random forest decision tree to predict a diagnosis using patients’ natural verbal complaints as user-generated data. In [20], the authors showed the results obtained by individual classification algorithms such as decision tree, random forest tree, and extra tree with an accuracy of 98%, 99%, and 93%, respectively.

### 2.3. Random Forest

The problem of imbalanced data in the medical field always exists. The study in [21] presented a misclassification synthetic minority over-sampling technique using a random forest for data resampling. It is often the case that random forest (RF) is frequently used in medical imagining and the timely detection of the risk factors that cause cancer or different abnormalities [22]. Moreover, RF is also used in the creation of AI smart monitoring systems, as shown in [23,24]. On the other hand, some studies have presented the results of using RF classifiers for predicting specific contagious and non-contagious diseases [25,26].

### 2.4. Artificial Neural Networks

One of the main objectives in study [27] was to propose an automated medical decision support system using the implementation of a convolutional neural network (CNN), or EfficientNet and 10-fold stratified cross-validation. Another study, [28], presented a heterogeneous modified artificial neural network (HMANN) for the early detection, segmentation, and diagnosis of chronic renal failure on the Internet of Medical Things (IoMT) platform. In the study presented in [29], the authors utilized multi-layer perceptron neural networks (MLP) and convolutional neural networks (CNN) to detect early signs of breast cell malignancies. Meanwhile, the authors in [30] conducted a comprehensive review of commonly employed CNNs in medical imaging processing, including AlexNet, GoogleNet, ResNet, R-CNN, and FCNN.

To the best of the authors’ knowledge, there are no similar studies using ANN architectures based on Taguchi’s orthogonal vector plans to predict a hyperinsulinemia diagnosis. Moreover, there are no research studies achieving better accuracy than the one obtained in this study.

## 3. Methodology

In order to achieve the main research goals, in this section, we will describe the following ML algorithms: naive Bayes, decision tree, random forest, and new models using different ANN architectures constructed according to Taguchi’s orthogonal vector plans.

### 3.1. Naïve Bayes

One of the probabilistic machine learning models that are used for classification tasks is called naive Bayes classifier. It is considered a simple but powerful algorithm for predictive modeling. The naive Bayes (NB) algorithm is based on Bayes’ theorem which provides a way to calculate the probability of a hypothesis given to our prior knowledge [31,32]. In this case, the training phase is fast because we only need the probability of every class and the probability of every class given different input (*x*) values to be calculated. It does not require coefficients that need to be fitted by optimization procedures. With a given NB model, it is possible to make predictions for new datasets using the Bayes theorem. The naive Bayes machine learning algorithm aims to identify the hypothesis with the maximum posterior probability (MAP) [33]. To represent this, the following formula is given (1):

(1) $$P\left(X|Y=c\right)=\frac{1}{\sqrt{2\pi \sigma_{c}^{2}}}\cdot e^{\frac{-{\left(x-\mu_{c}\right)}^{2}}{2\pi \sigma_{c}^{2}}}$$

It is possible to use the equation above to make predictions with real valued inputs. Calculating the error in this approach can be considered as the lowest possible test error rate in classification which is produced by any of the Bayes classifiers. Since, naive Bayes does not have any hyperparameters to tune, in the presented study, based on probability results, this classifier predicts the probability or share of each risk factor and total risk that leads to the development of hyperinsulinemia in adolescent age

### 3.2. Decision Tree

A decision tree is a type of supervised ML algorithm that can deal with both classification and regression problems, and is considered as the easiest algorithm to interpret and understand. The purpose of using decision trees is to create a training model that can be used to predict the class or value of a target variable by learning simple decision rules derived from previous (training) data [34,35]. Decision trees start at the tree’s root to predict class labels for records. Compare the value of the root attribute with the attributes of the record. Based on the comparison, follow the branch matching that value and jump to the next node. A decision tree algorithm uses a data structure called a tree to predict the outcome of a given problem. The decision tree model follows a supervised learning approach where a pre-processed dataset is utilized to train the algorithm. The tree structure is built with a top-down strategy, starting from the root node at the top and branching out to the tree leaves that represent the outcomes. The construction of the tree is accomplished through the use of a heuristic method known as recursive partitioning, which involves dividing the problem into smaller sub-problems until a satisfactory solution is found. The nodes that come after the root node are divided into many nodes [36]. The main concept is to divide the data space into dense and sparse regions using a decision tree. A binary tree can be split in two ways: binary or multi way. As long as the data is not sufficiently homogeneous, the method splits the tree repeatedly. A decision tree that can be utilized to generate the best-categorized predictions is returned at the conclusion of training. In this study, the parameters that will be used are:

1. max_depth: setting up the maximum depth in trees;
2. min_samples_split: minimum samples a node must contain to be available for a split;
3. min_samples_leaf: this controls the number of examples a terminal leaf node can have;
4. max_features: the number of features to consider when looking for the best split;
5. min_impurity_decrease: for controlling the amount of impurity, i.e., to define which splits are available.

### 3.3. Random Forest

One of the most widely used algorithms, from the supervised machine learning category, is definitely random forest. It consists of many decision trees, creating an algorithm that is trained through bagging or bootstrap aggregating. Bagging is an meta-algorithm that improves the exactness of machine learning algorithms. Like the name itself says, it has a large number of individual decision trees that operate as an ensemble [37,38]. Every decision tree in the random forest spits out a class forecast, and the classification that receives the most votes becomes the prediction made by the model. The key is the poor correlation between models. Uncorrelated models have the ability to provide ensemble forecasts that are more accurate than any of the individual predictions, just like assets with low correlations combine to build a portfolio that is larger than the sum of its parts. As long as they do not consistently all make a mistake in the same direction, the trees shield each other from their individual errors, which accounts for this result. Many trees will be right while some may be wrong, allowing the group of trees to travel in the proper direction [39]. Random Forests also offer a wider range of parameters that could be tuned. In this study, the focus will be on the following parameters:

1. n_estimators: the number of trees in the forest;
2. max_features: the number of features to consider when looking for the best split;
3. max_depth: the maximum depth of a tree;
4. criterion: the function to measure the quality of a split.

### 3.4. Experimental Setup—ANN-L(atin) Squares

Artificial neural networks, as powerful artificial intelligence tools, are increasingly used in medicine. They form a system of nodes or neurons interconnected by connections, through which data is transmitted. The architecture of any network consists of three parts: the input layer, the hidden layer, and the output layer. The input layer can have multiple sizes and inputs through which data is received. There may be one or more hidden layers, which are used to process data according to a given criterion, depending on the problem being solved [40]. An output layer can have one or more output values. The strength of the connection between neurons is called the weight factor. First, it is necessary to train the neural network and train it for further use. Our proposed model aims to select the most straightforward neural network architecture, with as few iterations as possible and minimal training, testing, and validation time. The main idea is to use a robust experiment design method based on Taguchi’s orthogonal vector plans [40,41]. Taguchi’s robust experimental design in each orthogonal plane depends on the number of parameters, the weighting coefficients, and the number of levels of each parameter. There are several plans for determining the dependence of the output and the input values through FFP (full factorial plan) when planning as many experiments as possible in which all possible discrete values of each input factor are combined [42,43]. When we have a large number of input factors (greater than 6), and at a large number of levels (greater than 5), then the number of experiments required is L^P^ (L is the number of levels of factor variation, and P is the factor number), that is, how many times is necessary to test each level for each parameter. The number of iterations required for a complete factorial analysis is N = *L^P^* (for example, when using three levels with 13 parameters according to a full factorial design, N = 3^13^ = 1,594,323 experiments need to be performed). Using a Taguchi orthogonal plan with 13 parameters (weight coefficients) at three levels, only orthogonal array = |27, 13, 3| = 3^3^ = 27 experiments are required. Taguchi’s robust design method reduces the number of experiments by 99.99830649% (0.9999830649 = 1 − (27/1594323)) [44]. Taguchi’s orthogonal vector plan takes a selected subset of combinations without repetition so that all parameters are considered equally. They can also be evaluated independently of each other. An orthogonal vector plan is observed for each level of a particular parameter. All L levels of each of the (P-1) other parameters are tested at least once [45,46]. 

**Figure 1:**
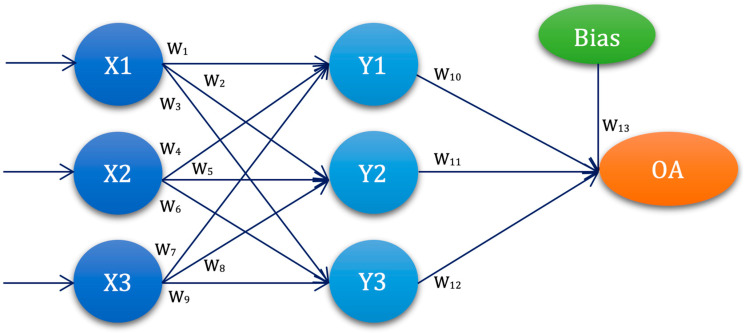
ANN-L27 architecture—graphical representation.

The first selected ANN architecture was with one hidden layer and three nodes, denoted as ANN-L27, with the corresponding orthogonal plan in Figure 1 and Table 1. 

The graphical representation of the ANN-L27 architecture in Figure 1 is constructed based on the L27 orthogonal plan, with three input values, one hidden layer with three nodes, and one output. ANN-L27 has a three-level architecture and thirteen weighting coefficients.

**Figure 2:**
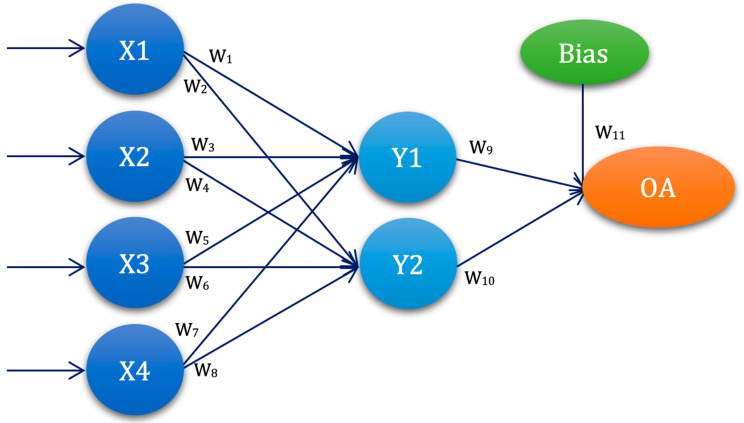
ANN-L12 architecture—graphical representation.

Th second used ANN architecture was with one hidden layer and two nodes, denoted as ANN-L12, with the corresponding orthogonal plan in Figure 2 and Table 2. 

The graphical representation of the ANN-L12 architecture in Figure 2 is constructed based on the L12 orthogonal plan, with four input values, one hidden layer with two nodes, and one output. ANN-L12 has a two-level architecture and eleven weighting coefficients.

**Figure 3:**
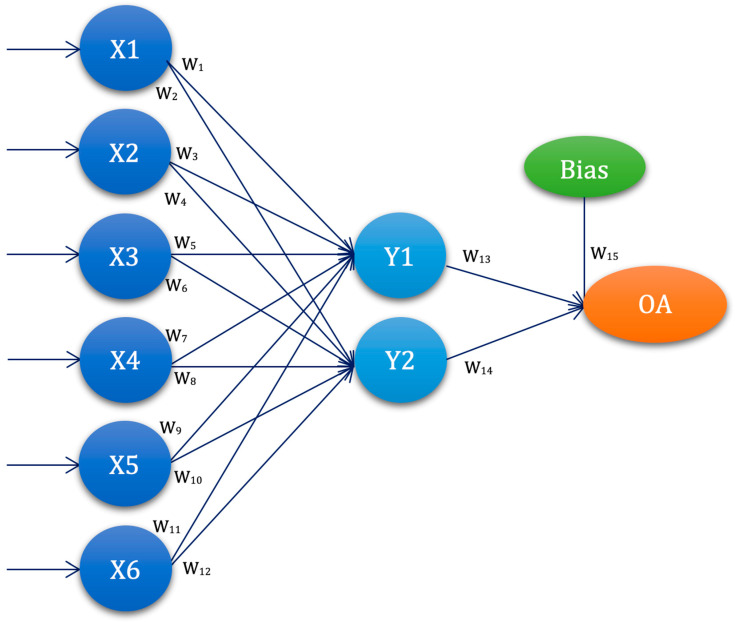
ANN-L16 architecture—graphical representation.

The third used ANN architecture was with one hidden layer and two nodes, denoted as ANN-L16, with the corresponding orthogonal plan Figure 3 and Table 3.

The graphical representation of the ANN-L16 architecture in Figure 3 is constructed based on the L16 orthogonal plan, with six input values, one hidden layer with two nodes, and one output. ANN-L16 has a two-level architecture and fifteen weighting coefficients.

Algorithm for robust design of the experimental part:

**Step 1:** Each of the three architectures used is a simple artificial neural network with one input layer. In the presented research, the input layer consists of three input risk factors for ANN-L27, four input risk factors for ANN-L12, and six risk factors for ANN-L16.

**Step 2:** The values of all investigated factors are represented by different values and measurement units. Therefore, it is necessary to translate them into coded values. In this way, all factors are equally represented and have the same influence on the risk of hyperinsulinemia. All input values are transformed according to the following formula: The function *μD*(*X*): R ⇒ [0, 1] translates the actual values of the input values into coded values from the interval [0, 1], as *μD*(*Y_i_*) = (*X_i_* − *X_min_*)/(*X_max_* − *X_min_*) [40,42]. *D* represents the data set on which the research is performed, *X_i_* is the input value, *X_min_* is the smallest input value, and *X_max_* is the maximum input value on the observed data set *D*.

**Step 3:** The sigmoid function was used as the activation function of the hidden and output layers, as shown in Formula (2):

(2) $$Y_{i}=\frac{1}{1+e^{-x_{i}}},\;i=\left(\bar{1,n}\right)$$

For example, the activation function used in Formula (3) for the ANN-L27 architecture is provided [42]:

$$Y_{1}=\frac{1}{1+e^{-\left(x_{1}W_{1}+x_{2}W_{4}+x_{3}W_{7}\right)}}$$

$$Y_{2}=\frac{1}{1+e^{-\left(x_{1}W_{2}+x_{2}W_{5}+x_{3}W_{8}\right)}}$$

$$Y_{3}=\frac{1}{1+e^{-\left(x_{1}W_{3}+x_{2}W_{6}+x_{3}W_{9}\right)}}$$

(3) $$OA\left(ANN-L27\right)=\frac{1}{1+e^{-\left(yW_{10}+y_{2}W_{11}+yW_{12}+1\cdot W_{13}\right)}}$$

In the first architecture of ANN-L27, an orthogonal plan with three levels, L1, L2, and L3, and initial values of weight factors Wi that take values from the interval [−1, 0, 1] is used. For each subsequent iteration, the values of the weighting factors are obtained by halving the interval, with the previous rejection of the highest value of the cost–effect function obtained from the first iteration. The second proposed architecture, ANN-L12, and the third architecture, ANN-L16, are constructed based on an orthogonal plan of two levels, L1, L2, and the initial value of the weight factor W*_i_* taking values from the interval [−1, 1]. For each subsequent iteration, new values of the weighting factors are calculated by halving the interval of the cost-effect function obtained in the first iteration. The cost–effect function is the total value of the relative error calculated according to the given orthogonal plan for the specified level.

For example, the value of the cost–effect function for the first listed ANN-L27 architecture is calculated using Formula (4) [42]:

(4) L1W_1_ = cost1 + cost2 +…+ cost9 L2W1 = cost10 + cost11 +…+ cost18 L3W1 = cost19 + cost20 +…+ cost27 … L1W_13_ = cost1 + cost5 +…+ cost26 L2W_13_ = cost2 + cost6 +…+ cost27 L3W_13_ = cost3 + cost4 +…+ cost25 if cost(i) = ∑MRE(ANN-L27(i))

**Step 4:** A decoding method is used in the following way (5), (6):

(5) $$Y_{i}=\left(X_{min}+\mu D\left(X_{i}\right)\right)\cdot \left(X_{max}-X_{min}\right)$$

(6) $$Risk\left(i\right)=OA\left(ANN_{i}\right)=\frac{1}{n}\sum_{i=1}^{n}Y_{i},\;and\;i=27,\;i=16,\;i=12$$

where *OA*(*ANN_i_*) represents the real risk, which is calculated according to ANN-L27, ANN-L12, and ANN-L16.

**Step 5:** For each iteration in this study, the output values are calculated according to formulas of the metrics presented below (7), (8):

(7) $$MRE=\frac{1}{n}\sum_{i=1}^{n}\left|ActEffort-EstEffort\right|$$

(8) MMRE=mean(MRE)

For each research part in each iteration, gradient descent (*GA*) is followed with the condition of *GA* < 0.01, calculated as (9):

(9) $$GA=MRE_{i1}-MRE_{i2}<0.01$$

where *i*(1, *n*) − *n* is the number of the architecture ANN.

The difference of minimum values for each iteration in each ANN architecture is denoted by delta(*i*) = *δ*, and is calculated as follows (10), (11):

(10) $$\delta_{i}=(OA\left(ANN_{k}-OA\left(ANN_{k-1}\right))\right)F_{m}$$

(11) $$if\;\delta_{i}>\delta_{\left(i+1\right)}\;then\;ANN_{i}\;is\;converging\;with\;MMRE_{i}$$

*i*—number of ANN, *k*—number of iterations, *m*—number of risk factors.

Hereby, in our research with different ANN architectures, we set the convergence-stopping criterion (number of iterations) to *GA* < 0.01. In the training phase of the selected ANN architecture according to Taguchi’s orthogonal plan, in each subsequent iteration, a reduction in MRE of less than 1% is achieved, which in our experiment represents the “stopping criterion” [40,42].

**Step 6:** Examining the impact of the input values on the change in risk factor values:

1. The effect of the first input factor (BMI) and its value is calculated as:

(12) $$\begin{array}{c} \delta 1=mean\left(OA\left(ANN_{k}\right)\right)-mean\left(OA\left(ANN_{k-1}\right)\right)F1\\ if\;\left(OA\left(ANN_{k}\right)\right)F1,\;mean\left(OA\left(ANN_{k-1}\right)\right)F1\\ then\;X1=0;X1=BMI. \end{array}$$

2. The effect of the second input factor (*Cholesterol*) and its value is calculated as:

(13) $$\begin{array}{c} \delta 2=mean\left(OA\left(ANN_{k}\right)\right)-mean\left(OA\left(ANN_{k-1}\right)\right)F\\ if\;\left(OA\left(ANN_{k}\right)\right)F2,\;mean\left(OA\left(ANN_{k-1}\right)\right)F2\\ then\;X2=0;X2=Cholesterol. \end{array}$$

3. The effect of the third input factor (*Physical activity*) and its value is calculated as:

(14) $$\begin{array}{c} \delta 3=mean\left(OA\left(ANN_{k}\right)\right)-mean\left(OA\left(ANN_{k-1}\right)\right)F\\ if\;\left(OA\left(ANN_{k}\right)\right)F3,\;mean\left(OA\left(ANN_{k-1}\right)\right)F3\\ then\;X3=0;X3=Physical\;activity. \end{array}$$

4. The effect of the fourth input factor (*Family history*) and its value is calculated as:

(15) $$\begin{array}{c} \delta 4=mean\left(OA\left(ANN_{k}\right)\right)-mean\left(OA\left(ANN_{k-1}\right)\right)F4\\ if\;\left(OA\left(ANN_{k}\right)\right)F4,\;mean\left(OA\left(ANN_{k-1}\right)\right)F4\\ then\;X4=0;X4=Family\;history. \end{array}$$

**Step 7:** Calculating the values of Pearson’s and Spearman’s correlation coefficients [43,44,46].

### 3.5. Dataset Description

The research population consisted of adolescents of both genders, aged 12 to 17 years from the territory of the Kolubara district, who came for a regular, systematic examination at the Valjevo Health Center, as a reference health institution in this field, in the period from September 2019 to September 2022, and in whom elevated glycemic values were verified. Respondents were included in the study with their voluntary informed consent, that is, the consent of their parents, taken after familiarization with the study orally and in writing, as well as after signing the form for informed consent of the respondents. This research was approved by the Ethics Committee of Valjevo Health Center (latest/renewed decision DZ-01-2646 dated 9 August 2021). Sampling and then a grouping of patients was conducted based on authoritative guidelines for defining the presence of hyperinsulinemia in adolescents [47] whom the pediatrician instructed to perform an OGTT during a systematic school examination due to elevated glycemic values. The experimental group of patients consisted of adolescents who, during the implementation of the OGTT, had an insulinemia value of >15 μU/mL after and/or during the 120 min of the OGTT of >75 μU/mL. The control group consisted of adolescents who, during the OGTT, had insulinemia values of ≤15 μU/mL at the end, i.e., ≤75 μU/mL during the 120 min. The first experimental group comprised 112 male and female adolescents, and the second control group comprised 224 male and female adolescents. Independent and confounding variables were collected using relevant standardized questionnaires in this field that were free to use, such as the Child Health Questionnaire (CHQ) [48]— the world standardized questionnaire for the assessment of physical and psychosocial well-being; the International Physical Activity Questionnaires (IPAQ) [49]—a standardized physical activity assessment questionnaire; Association for Sports and Sports Medicine, Ministry of Youth and Sports of the Republic of Serbia, Youth/Adolescent food questionnaire (YAQ) [50]—a standardized questionnaire for high school students, which collects information about habits in nutrition; Behavioral Risk Factor Surveillance System survey (BRFSS) [51]—a standardized survey on the risk assessment of chronic non-communicable diseases, which contains information on health status, chronic conditions, alcohol consumption and similar; Family history questionnaire (FHQ) [52]—a family history questionnaire; and Short form health survey-6 (SF-36) [53]—a standardized questionnaire for assessing the quality of life of adolescents. Primary data such as demographic characteristics of the respondent, including the gender, age of the patient, and socio-economic conditions of the respondent, the environment from which he comes (urban, suburban or rural), the number of household members, study conditions, and place of residence and living conditions (with parents, tenant, relatives, others) were collected in the first phase of the research when coming for a systematic examination of adolescents, and before filling out the other questionnaires. An overview of the sample size according to gender, age and Kolubara district is given in Table 4.

### 3.6. Statistical Analysis

According to the obtained analysis it can be concluded that increased obesity, that is, the value of the body mass index, is significantly higher in the experimental group compared to the control group, which is a significant indicator of the cause of hyperinsulinemia in adolescents with hyperglycemia. The average BMI in the experimental group is 27.1 with a deviation of ±4.3, while in the control group, it is within the limits of typical values and is 22.7 with a deviation of ±1.2. Table 5 shows the average glucose and insulin values of all respondents for the mentioned groups. The specified values were monitored at 0, 30, 60, 90, and 120 min. Then, the mean values with deviations for the respondents of each group were calculated. Based on the results obtained from the mentioned measurements and according to Formula (16), the insulin resistance index HOMA-IR values were calculated [53]. Based on the analysis with the student *t*-test, the values obtained are statistically significant between the experimental groups, which once again confirms the correctness of the division into given groups based on the OGTT test. From all of the above, it can be concluded that the insulin resistance index, HOMA-IR, is a reliable predictor of the diagnosis of hyperinsulinemia in the adolescent population.

(16) $$\mathrm{HOMA}-\mathrm{IR}=\frac{\mathrm{Glucose}\left(0\min\right)\cdot \mathrm{Insuline}\left(0\min\right)}{22.5}$$

In Table 6, the values were also analyzed according to the gender of the respondents within each group to determine their differences. The Kruskal–Wallis H test shows statistically significant differences in blood count values, leukocytes, erythrocytes, hemoglobin, and hematocrit. After that, there are statistically significant differences in the respondents of the first and control groups regarding platelets, lymphocytes, and sedimentation; CRP values are elevated. The respondents were instructed to complete the OGTT test based on elevated glucose. The Kruskal–Wallis H test shows highly significant differences in the values of total cholesterol, HDL cholesterol, LDL cholesterol, and triglycerides, which indicates the obesity of the respondents, which, in addition to the development and occurrence of hyperinsulinemia, can also lead to the development of many other non-infectious chronic diseases. The values of urea, creatinine, total proteins, total bilirubin, AST(SGOT) and ALT(SGPT), and sodium, potassium, and chloride are outside the reference values with statistically significant differences between the groups. 

**Figure 4:**
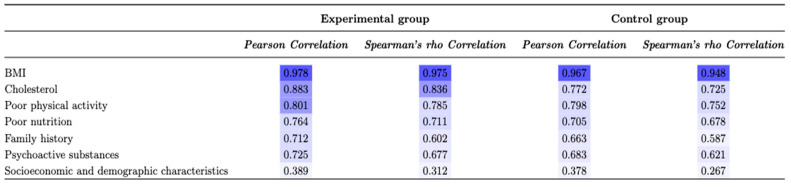
Correlation coefficients between the risk factors.

Based on all the listed values of the hematological and biochemical parameters and the OGTT test values, the following factors can be identified, as given in Table 7. Furthermore, in Figure 4, the correlation coefficients between the most influential risk factors from experimental and control groups are given.

## 4. Results

In this section, we present and discuss the results of the parameter tuning with grid search and cross-validation assessment on the predictive performance of the models. The first part presents the results obtained by factorial analysis. In the second part, we show the results obtained from three ML algorithms. The third part is devoted to results obtained by the new ANN-L model. Finally, the last part gives a comparison of acquired results.

### 4.1. Factorial Analysis

Factor analysis is a statistical method that aims to group a large number of similar variables around one or more of those variables that best describe a given characteristic or a particular influence, a factor. First, a factorial exploratory analysis was used to separate the two most influential factors: BMI with a 35.8% and cholesterol with a 15.3% share in the total risk for hyperinsulinemia. Then, similarly, using confirmatory factor analysis, four more significant factors were singled out, namely poor physical activity at 14.1%, poor nutrition at 12.0%, family history at 9.0%, and the consumption of psychoactive substances at 7.1%. Other factors observed have a 6.7% share in the risk of hyperinsulinemia with elevated glycemia.

### 4.2. Naïve Bayes, Decision Tree, and Random Forest

**Figure 5:**
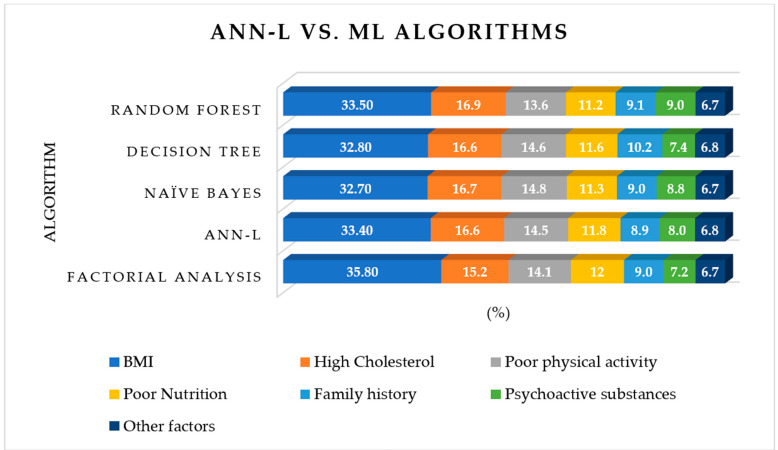
Graphical representation—the percentage share of each risk factor for the occurrence of hyperinsulinemia for the used models.

The state-of-the-art machine learning algorithms were used to identify risk factors in the overall risk of hyperinsulinemia. The results obtained using the first, the naive Bayes machine learning algorithm, showed the following prediction percentages: BMI with 32.7%, cholesterol with 16.7%, poor physical activity with 14.8%, poor nutrition with 11.3%, family history with 9.0%, consumption of psychoactive substances with 8.8% and other factors with a share of 6.7% of the total risk. The second, the decision tree machine learning algorithm showed the following prediction percentages: BMI with 32.8%, cholesterol with 16.6%, poor physical activity with 14.6%, poor nutrition with 11.6%, family history with 10.2%, consumption of psychoactive substances with 7.4% and other factors with a share of 6.8% of the total risk. The third algorithm used, the random forest machine learning algorithm showed the following prediction percentages: BMI with 33.5%,cholesterol with 16.9%, poor physical activity with 13.6%, poor nutrition with 11.2%, family history with 9.1%, consumption of psychoactive substances with 9.0% and other factors with a share of 6.7% of the total risk (Figure 5).

The graphical representation from Figure 5 showcases the proportionate contribution of each risk factor to the incidence of hyperinsulinemia, as determined by the models employed.

### 4.3. ANN Based on Taguchi’s Orthogonal Vector Plans (ANN-L)

**Figure 6:**
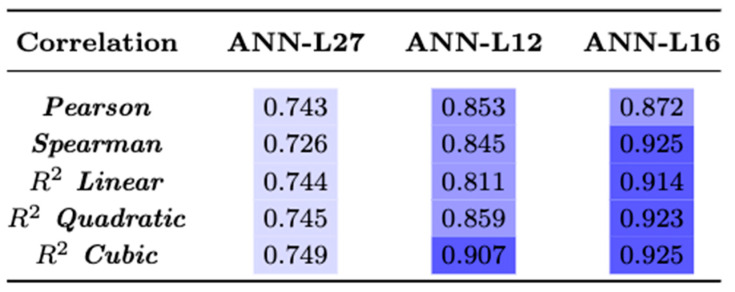
Correlation coefficients—ANN-L.

**Figure 7:**
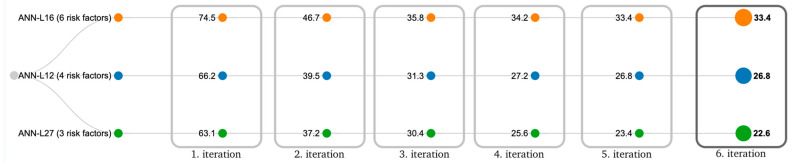
Monitoring of the value of the total risk for the occurrence of hyperinsulinemia within the framework of all three proposed ANN-L models (ANN-L12, ANN-L16, ANN-L27), on the total sample through six iterations.

The findings obtained from the proposed ANN-L model will be presented in detail. In the first experiment, the ANN-L27 architecture was constructed based on Taguchi’s orthogonal vector plan L27. The three input variables used in our example are the three most influential risk factors: BMI, cholesterol, and poor physical activity. These three risk factors had 22.6% of the total risk, and the error that occurred was less than 1%, which was the condition for stopping the number of iterations (GA < 0.01). It was necessary to perform less than six iterations to complete this experiment. In the second experiment, the ANN-L12 architecture was constructed based on Taguchi’s orthogonal vector plan L12. The four input variables used in our example are the four most influential risk factors: BMI, cholesterol, poor physical activity, and poor nutrition. These four risk factors had 26.8% of the total risk, and the error that occurred was less than 1%, which was the condition for stopping the number of iterations (GA < 0.01). In the third experiment, the ANN-L16 architecture was constructed based on Taguchi’s orthogonal vector plan L16. The six input variables used in our example are the six most influential risk factors: BMI, cholesterol, poor physical activity, poor nutrition, family history, and psychoactive substances. These six risk factors had 33.4% of the total risk, and the error that occurred was less than 1%, which was the condition for stopping the number of iterations (GA < 0.01). The correlation between the estimated and actual values for all three architectures: ANN-L27, ANN-L12 and ANN-L16 is given in Figure 6. The correlation coefficients have a higher value, which is another indicator of the precision and reliability of the artificial neural networks used in the experimental part. We conclude that the ANN- L16 architecture has the highest values of all correlation coefficients and the smallest number of iterations for which it meets the GA criterion (error less than 1%), with only five iterations required (Figure 7).

### 4.4. Comparative Analysis of the Models

**Figure 8:**
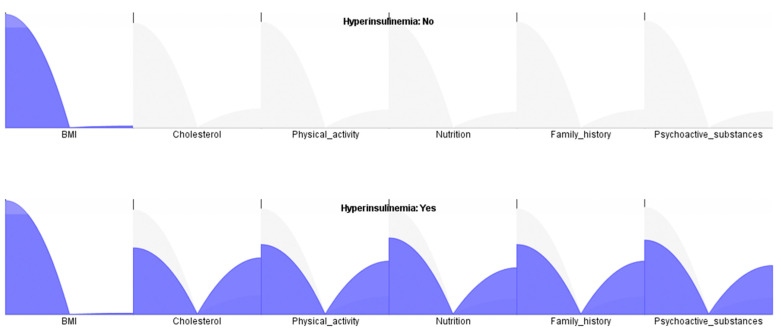
Graphical representation—occurrence of hyperinsulinemia within the risk factors assessed by ANN-L—experimental and control group.

In this section, we will present the results of the various models utilized in this study and conduct a thorough comparison to identify the most reliable and practical model for implementation. From Table 8, it can be concluded that the factor with the largest share of the total risk is BMI, with the fact that in the factorial analysis, it is 35.8%, and in the naive Bayes algorithm, it shows the lowest value of 32.7%. The share of all factors is ranked equally. Additionally, we can conclude, e.g., that the factor with the smallest share of the total risk is in the interval of 7.2% with factorial analysis. In comparison, its highest value is 9.0% with the random forest algorithm. Figure 8 is a graphical representation of the influencing factors for the occurrence of hyperinsulinemia in adolescents. Moreover, we can conclude when hyperinsulinemia is presented in the experimental and control group. Furthermore, it can be concluded that the best MMRE is acquired with the new, proposed ANN-L model (0.5%), which means that model accuracy is 99.5%. The second best result was achieved with the random forest algorithm (0.8%), which provides a model accuracy of 99.2%. The naive Bayes algorithm had a slightly worse model accuracy of 99.1%, while decision tree and factorial analysis showed model errors of 1.1% and 1.3%, which contributes to a model accuracy of 98.9% and 98.7%, respectively (Table 8).

## 5. Discussion

Metabolic syndromes, such as insulin resistance and hyperinsulinemia, most often results in type 2 diabetes mellitus, or cause the development of various cardiovascular diseases, dyslipidemia, and other serious non-contagious diseases. One of the components of severe chronic diseases, hyperinsulinemia, is the most common criterion for developing type 2diabetes mellitus, according to the International Diabetes Federation [53]. 

In order to achieve the first primary objective of the study, which is to identify the most influential factors for the occurrence of hyperinsulinemia in adolescents, the following conclusions can be drawn: The minimization of the estimation error (MMRE) to 0.5% was achieved using the newly proposed ANN-L models. This demonstrates a high accuracy of 99.5%. Among the selected ANN-L models, if three risk factors are considered, the total risk of receiving a hyperinsulinemia diagnosis using ANN-L27 is 22.6%. On the other hand, if four risk factors are considered using ANN-L12, the total risk of receiving a hyperinsulinemia diagnosis is 26.8%. Meanwhile, if six risk factors are considered using ANN-L16, the total risk of receiving a hyperinsulinemia diagnosis is 33.4%. Additionally, the number of iterations performed for ANN-L27 was six, while for ANN-L12 and ANN-L16 it was five, which resulted in a high convergence rate and faster evaluation. Faster evaluation in medical diagnostics is of great importance as it leads to more straightforward and precise results. Finally, the ANN-L16 model achieved the lowest error rate of 0.5% with the lowest number of iterations performed.

The second main goal of this study aimed to determine which of the presented models using different machine learning algorithms and artificial neural network architectures based on different Taguchi’s orthogonal vector plans would be the most accurate in determining the risk of hyperinsulinemia in adolescents. The results of the study indicated that the three most common factors contributing to the risk of hyperinsulinemia in adolescents are increased body mass index (35.8%), increased cholesterol levels (15.2%), and poor physical activity (14.1%). The remaining factors, including poor nutrition, family history, and consumption of psychoactive substances, also have a significant impact on the risk of hyperinsulinemia. The study showed that these six factors were the most influential in the development of hyperinsulinemia in adolescents, while other demographic and socioeconomic conditions had a smaller impact. All models used in this study demonstrated that these six factors are crucial in determining the risk of hyperinsulinemia in adolescents.

## 6. Conclusions

Given that the prevalence of hyperinsulinemia in adolescents, both in the world and in our country, is growing rapidly, the results of this study can be of exceptional scientific and practical importance to pediatricians. Furthermore, they can help in this field by creating a strategy for applying preventive and timely corrective measures to prevent the occurrence of the mentioned pathophysiological entity, that is, the development of potential complications (primarily type 2 diabetes mellitus and cardiovascular diseases) in later adult life. The results of this research can significantly contribute to a better knowledge and understanding of risk factors that can significantly affect the occurrence of hyperinsulinemia with elevated glycemia in adolescents, especially for those adolescents who have increased obesity, bad eating habits, insufficient physical activity, the existence of a positive family history, and the consumption of psychoactive drugs substances. The innovativeness of the proposed approach is reflected in the fact that, unlike other models that use machine learning algorithms, here it is possible to create a prediction model that is based on ANNs created based on Taguchi’s orthogonal vector plans in such a way as to achieve the lowest MMRE value in all phases of the study. Additionally, the aim was to create the most straightforward architecture, with the smallest number of hidden layers of the feed-forward artificial neural network and the smallest number of iterations, which additionally reduced the estimation time, which indeed enables timely and fast diagnosis, which is extremely important in medical sciences. The early detection of adolescent individuals who are prone to developing hyperinsulinemia is crucial for ensuring their future well-being and the overall health of society. The proposed models serve as a reliable tool for identifying the risk factors associated with hyperinsulinemia and other health issues that may negatively impact the individual’s well-being. By providing accurate and timely information, these models have the potential to play a critical role in preventing the development of hyperinsulinemia and mitigating its associated health risks.

## Figures and Tables

**Table 1:** ANN-L27 orthogonal vector plan.

ANN-L27	W_1_	W_2_	W_3_	W_4_	W_5_	W_6_	W_7_	W_8_	W_9_	W_10_	W_11_	W_12_	W_13_
ANN1	L1	L1	L1	L1	L1	L1	L1	L1	L1	L1	L1	L1	L1
ANN2	L1	L1	L1	L1	L2	L2	L2	L2	L2	L2	L2	L2	L2
ANN3	L1	L1	L1	L1	L3	L3	L3	L3	L3	L3	L3	L3	L3
ANN4	L1	L2	L2	L2	L1	L1	L1	L2	L2	L2	L3	L3	L3
ANN5	L1	L2	L2	L2	L2	L2	L2	L3	L3	L3	L1	L1	L1
ANN6	L1	L2	L2	L2	L1	L1	L1	L3	L3	L3	L2	L2	L2
ANN7	L1	L3	L3	L3	L1	L1	L1	L3	L3	L3	L2	L2	L2
ANN8	L1	L3	L3	L3	L2	L2	L2	L1	L1	L1	L3	L3	L3
ANN9	L1	L3	L3	L3	L3	L3	L3	L2	L2	L2	L1	L1	L1
ANN10	L2	L1	L2	L3	L1	L2	L3	L1	L2	L3	L1	L2	L3
ANN11	L2	L1	L2	L3	L2	L3	L1	L2	L3	L1	L2	L3	L1
ANN12	L2	L1	L2	L3	L3	L1	L2	L3	L1	L2	L3	L1	L2
ANN13	L2	L2	L3	L1	L1	L2	L3	L2	L3	L1	L3	L1	L2
ANN14	L2	L2	L3	L1	L2	L3	L1	L3	L1	L2	L1	L2	L3
ANN15	L2	L2	L3	L1	L3	L1	L2	L1	L2	L3	L2	L3	L1
ANN16	L2	L3	L1	L2	L1	L2	L3	L3	L1	L2	L2	L3	L1
ANN17	L2	L3	L1	L2	L2	L3	L1	L1	L2	L3	L3	L1	L2
ANN18	L2	L3	L1	L2	L3	L1	L2	L2	L3	L1	L1	L2	L3
ANN19	L3	L1	L3	L2	L1	L3	L2	L1	L3	L2	L1	L3	L2
ANN20	L3	L1	L3	L2	L2	L1	L3	L2	L1	L3	L2	L1	L3
ANN21	L3	L1	L3	L2	L3	L2	L1	L3	L2	L1	L3	L2	L1
ANN22	L3	L2	L1	L3	L1	L3	L2	L2	L1	L3	L3	L2	L1
ANN23	L3	L2	L1	L3	L2	L1	L3	L3	L2	L1	L1	L3	L2
ANN24	L3	L2	L1	L3	L3	L2	L1	L1	L3	L2	L2	L1	L3
ANN25	L3	L3	L2	L1	L1	L3	L2	L3	L2	L1	L2	L1	L3
ANN26	L3	L3	L2	L1	L2	L1	L3	L1	L3	L2	L3	L2	L1
ANN27	L3	L3	L2	L1	L3	L2	L1	L2	L1	L3	L1	L3	L2

**Table 2:** ANN-L12 orthogonal vector plan.

ANN-L12	W_1_	W_2_	W_3_	W_4_	W_5_	W_6_	W_7_	W_8_	W_9_	W_10_	W_11_
ANN1	L1	L1	L1	L1	L1	L1	L1	L1	L1	L1	L1
ANN2	L1	L1	L1	L1	L1	L2	L2	L2	L2	L2	L2
ANN3	L1	L1	L2	L2	L2	L1	L1	L1	L2	L2	L2
ANN4	L1	L2	L1	L2	L2	L1	L2	L2	L1	L1	L2
ANN5	L1	L2	L2	L1	L2	L2	L1	L2	L1	L2	L1
ANN6	L1	L2	L2	L2	L1	L2	L2	L1	L2	L1	L1
ANN7	L2	L1	L2	L2	L1	L1	L2	L2	L1	L2	L1
ANN8	L2	L1	L2	L1	L2	L2	L2	L1	L1	L1	L2
ANN9	L2	L1	L1	L2	L2	L2	L1	L2	L2	L1	L1
ANN10	L2	L2	L2	L1	L1	L1	L1	L2	L2	L1	L2
ANN11	L2	L2	L1	L2	L1	L2	L1	L1	L1	L2	L2
ANN12	L2	L2	L1	L1	L2	L1	L2	L1	L2	L2	L1

**Table 3:** ANN-L16 orthogonal vector plan.

ANN-L16	W_1_	W_2_	W_3_	W_4_	W_5_	W_6_	W_7_	W_8_	W_9_	W_10_	W_11_	W_12_	W_13_	W_14_	W_15_
ANN1	L1	L1	L1	L1	L1	L1	L1	L1	L1	L1	L1	L1	L1	L1	L1
ANN2	L1	L1	L1	L1	L1	L1	L1	L2	L2	L2	L2	L2	L2	L2	L2
ANN3	L1	L1	L1	L2	L2	L2	L2	L1	L1	L1	L1	L2	L2	L2	L2
ANN4	L1	L1	L1	L1	L2	L2	L2	L2	L2	L2	L2	L1	L1	L1	L1
ANN5	L1	L2	L2	L1	L1	L2	L2	L1	L1	L2	L2	L1	L1	L2	L2
ANN6	L1	L2	L2	L1	L1	L2	L2	L2	L2	L1	L1	L2	L2	L1	L1
ANN7	L1	L2	L2	L2	L2	L1	L1	L1	L1	L2	L2	L2	L2	L1	L1
ANN8	L1	L2	L2	L2	L2	L1	L1	L2	L2	L1	L1	L1	L1	L2	L2
ANN9	L2	L1	L2	L1	L2	L1	L2	L1	L2	L1	L2	L1	L2	L1	L2
ANN10	L2	L1	L2	L1	L2	L1	L2	L2	L1	L2	L1	L2	L1	L2	L1
ANN11	L2	L1	L2	L2	L1	L2	L1	L1	L2	L1	L2	L2	L1	L2	L1
ANN12	L2	L1	L2	L2	L1	L2	L1	L2	L1	L2	L1	L1	L2	L1	L2
ANN13	L2	L2	L1	L1	L2	L2	L1	L1	L2	L2	L1	L1	L2	L2	L1
ANN14	L2	L2	L1	L1	L2	L2	L1	L2	L1	L1	L2	L2	L1	L1	L2
ANN15	L2	L2	L1	L2	L1	L1	L2	L1	L2	L2	L1	L2	L1	L1	L2
ANN16	L2	L2	L1	L2	L1	L1	L2	L2	L1	L1	L2	L1	L2	L2	L1

**Table 4:** Structure of the dataset used.

Sample Structure				
Experimental Group		Control Group		
Gender	Number	Percentage(%)	Number	Percentage(%)
male	108	48.2	228	50.9
female	116	51.8	220	49.1
Total	224	100.0	448	100.0
Age	Number	Percentage(%)	Number	Percentage(%)
12–14	108	48.2	228	50.9
14–17	116	51.8	220	49.1
Total	224	100.0	448	100.0
Region	Number	Percentage(%)	Number	Percentage(%)
Kolubara district	224	100.0	448	100.0

**Table 5:** Values of OGTT and HOMA-IR.

OGTT	Experimental Group	Control Group	Student *t* Test	*p*
Glucose in 0 min (mmol/L)	7.2 ± 1.1	6.3 ± 0.9	2.026	0.026 *
Glucose in 30 min (mmol/L)	13.5 ± 1.3	11.3 ± 1.4	2.844	0.006 *
Glucose in 60 min (mmol/L)	10.7 ± 1.4	9.3 ± 1.2	5.124	0.000 *
Glucose in 90 min (mmol/L)	9.4 ± 1.3	8.2 ± 1.3	2.895	0.008 *
Glucose in 120 min (mmol/L)	8.1 ± 0.7	7.3 ± 0.9	2.387	0.017 *
Insuline in 0 min (μIU/mL)	20.3 ± 3.6	17.8 ± 2.4	7.264	0.000 *
Insuline in 30 min (μIU/mL)	162.5 ± 6.1	151.2 ± 7.1	118.371	0.000 *
Insuline in 60 min (μIU/mL)	125.7 ± 4.5	117.3 ± 5.2	84.625	0.000 *
Insuline in 90 min (μIU/mL)	98.3 ± 2.2	83.5 ± 3.7	81.814	0.000 *
Insuline in 120 min (μIU/mL)	83.5 ± 3.4	65.3 ± 2.4	6.078	0.000 *
HOMA-IR	6.5 ± 2.4	5.2 ± 1.7	4.680	0.000 *

* Statistical significance.

**Table 6:** Values of hematological and biochemical parameters measured in both groups.

Gender	Experimental Group		Control Group		Kruskal–Wallis H	*p*
	Male	Female	Male	Female		
Leukocytes WBC	14.3 ± 2.7	16.8 ± 3.5	11.5 ± 2.4	12.7 ± 3.3	13.322	0.001 *
Erythrocytes RBC	3.5 ± 1.5	3.7 ± 2.1	4.2 ± 2.5	4.5 ± 2.6	10.956	0.004 *
Hemoglobin Hgb	156 ± 5	145 ± 7	138 ± 5	142 ± 4	5.735	0.017 *
Hematocrit Htc	0.626 ± 0.6	0.548 ± 0.9	0.533 ± 0.5	0.427 ± 0.7	4.725	0.030 *
MCV	98.4 ± 10.3	92.5 ± 11.7	89.1 ± 12.2	94.2 ± 9.4	0.997	0.318
MCH	36.7 ± 3.3	34.5 ± 4.5	33.2 ± 2.1	33.5 ± 3.6	2.735	0.098
MCHC	358.9 ± 17.6	345.2 ± 18.9	344.1 ± 12.3	338.7 ± 15.7	0.525	0.769
RDW	17.2 ± 2.7	16.7 ± 3.1	16.3 ± 2.8	15.9 ± 3.6	1.925	0.165
Platelets PLT	324.2 ± 67.2	345.6 ± 84.4	318.9 ± 58.3	338.2 ± 62.4	12.023	0.003 *
Segmented	54 ± 6.8	57 ± 7.9	52 ± 6.2	55 ± 7.5	0.752	0.386
MID	9.8 ± 1.5	10.2 ± 2.4	8.7 ± 1.4	9.3 ± 1.9	0.851	0.356
Lymphocytes	32.3 ± 3.3	31.7 ± 3.8	31.5 ± 3.1	33.6 ± 3.5	3.847	0.043 *
Sedimentation	17.8 ± 2.2	18.2 ± 2.5	15.4 ± 1.8	14.8 ± 2.4	4.205	0.036 *
CRP	18.3 ± 5.3	21.5 ± 6.7	16.3 ± 4.2	14.7 ± 77.9	149.599	0.000 *
Glucose	7.6 ± 1.6	8.4 ± 2.6	6.7 ± 1.3	7.3 ± 1.8	4.829	0.024 *
Cholesterol	7.11 ± 4.2	8.27 ± 5.4	5.93 ± 3.6	6.08 ± 4.4	87.774	0.000 *
HDL Cholesterol	0.723 ± 0.5	0.845 ± 0.7	0.994 ± 0.6	0.805 ± 0.6	73.497	0.000 *
LDL Cholesterol	3.58 ± 1.2	3.92 ± 1.7	3.23 ± 1.3	3.31 ± 1.5	55.961	0.000 *
Triglycerides	3.71 ± 2.8	4.26 ± 3.4	2.87 ± 2.6	2.99 ± 3.3	23.980	0.000 *
Urea	10.4 ± 3.2	12.5 ± 4.3	8.3 ± 2.5	9.7 ± 3.6	5.024	0.018 *
Creatinine	112.6 ± 11.5	115.8 ± 14.3	108.6 ± 9.6	114.7 ± 10.0	4.527	0.027 *
Proteins total	94.3 ± 3.5	102.7 ± 4.4	85.7 ± 3.2	89.2 ± 3.3	8.323	0.007 *
Bilirubin total	19.8 ± 4.3	23.7 ± 5.2	18.3 ± 3.8	19.5 ± 4.1	6.024	0.016 *
AST(SGOT)	35.6 ± 3.2	38.4 ± 3.9	33.8 ± 2.8	35.4 ± 3.1	4.418	0.023 *
ALT(SGPT)	39.6 ± 4.5	44.7 ± 5.7	35.2 ± 3.6	37.9 ± 4.2	6.134	0.019 *
Sodium	152 ± 14.2	165 ± 16.3	148 ± 11.5	159 ± 13.8	4.324	0.031 *
Potassium	5.3 ± 1.4	5.9 ± 1.9	5.1 ± 1.2	5.7 ± 1.3	5.235	0.024 *
Chlorides	111 ± 9	114 ± 13	105 ± 9	109 ± 11	7.456	0.012 *

* Statistical significance.

**Table 7:** Analysis of parameters that indicate the existence of hyperinsulinemia.

Parameters	Experimental Group Mean ± SD N(%)	Control Group Mean ± SD N(%)	ANOVA $\mathrm{Kruskal}–\mathrm{Wallis}\;\chi^{2}\mathrm{Test}$	*p*
Body height	160.6 ± 13.2	157.6 ± 12.5	2.841	0.032 *
Body weight	69.8 ± 11.4	56.4 ± 8.2	3.269	0.005 *
BMI	27.1 ± 4.3	22.7 ± 1.2	3.841	0.003 *
Cholesterol	87 (77.7)	129 (57.6)	8.645	0.000 *
Poor physical activity	78 (69.6)	136 (60.7)	2.0158	0.021 *
Poor nutrition	65 (58.0)	102 (45.6)	3.040	0.020 *
Family history	55 (49.1)	93 (41.5)	4.335	0.027 *
Psychoactive substances	43 (38.4)	78 (34.8)	2.013	0.031 *
Socioeconomic and demographic characteristics	27 (24.1)	51 (22.8)	1.492	0.221
Self-assessment of one’s own health condition	55 (49.1)	123 (54.9)	3.812	0.018 *

* Statistical significance.

**Table 8:** The percentage share of each risk factor in the used models.

Risk Factors	Factorial Analysis	ANN-L	Naïve Bayes	Decision Tree	Random Forest
BMI	35.8	33.4	32.7	32.8	33.5
High Cholesterol	15.3	16.6	16.7	16.6	16.9
Poor physical activity	14.1	14.5	14.8	14.6	13.6
Poor Nutrition	12.0	11.8	11.3	11.6	11.2
Family history	9.0	8.9	9.0	10.2	9.1
Psychoactive substances	7.2	8.0	8.8	7.4	9.0
Other factors	6.7	6.8	6.7	6.8	6.7
**MMRE**	**1.3%**	**0.5%**	**0.9%**	**1.1%**	**0.8%**

## Data Availability

The raw dataset used for this study is under a Non-Disclosure Agreement (NDA) and is therefore not available to the public.
